# Controversies about sugars: results from systematic reviews and meta-analyses on obesity, cardiometabolic disease and diabetes

**DOI:** 10.1007/s00394-016-1345-3

**Published:** 2016-11-30

**Authors:** Tauseef A. Khan, John L. Sievenpiper

**Affiliations:** 1 Department of Nutritional Sciences, Faculty of Medicine, University of Toronto, Toronto, ON Canada; 2 Toronto 3D Knowledge Synthesis and Clinical Trials Unit, Clinical Nutrition and Risk Factor Modification Centre, St. Michael’s Hospital, Toronto, ON Canada; 3 Division of Endocrinology and Metabolism, St. Michael’s Hospital, Toronto, ON Canada; 4 Li Ka Shing Knowledge Institute, St. Michael’s Hospital, Toronto, ON Canada

**Keywords:** Sugars, Fructose, Obesity, Overweight, Diabetes, Cardiovascular disease, Review

## Abstract

Fructose-containing sugars are a focus of attention as a public health target for their putative role in obesity and cardiometabolic disease including diabetes. The fructose moiety is singled out to be the primary driver for the harms of sugars due to its unique endocrine signal and pathophysiological role. However, this is only supported by ecological studies, animal models of overfeeding and select human intervention studies with supraphysiological doses or lack of control for energy. The highest level of evidence from systematic reviews and meta-analyses of controlled trials has not shown that fructose-containing sugars behave any differently from other forms of digestible carbohydrates. Fructose-containing sugars can only lead to weight gain and other unintended harms on cardiometabolic risk factors insofar as the excess calories they provide. Prospective cohort studies, which provide the strongest observational evidence, have shown an association between fructose-containing sugars and cardiometabolic risk including weight gain, cardiovascular disease outcomes and diabetes only when restricted to sugar-sweetened beverages and not for sugars from other sources. In fact, sugar-sweetened beverages are a marker of an unhealthy lifestyle and their drinkers consume more calories, exercise less, smoke more and have a poor dietary pattern. The potential for overconsumption of sugars in the form of sugary foods and drinks makes targeting sugars, as a source of excess calories, a prudent strategy. However, sugar content should not be the sole determinant of a healthy diet. There are many other factors in the diet—some providing excess calories while others provide beneficial nutrients. Rather than just focusing on one energy source, we should consider the whole diet for health benefits.

## Introduction

Overconsumption of dietary sugars with their potential to cause cardiometabolic disease has emerged as an important public health issue, highlighted by the vast coverage given in academic journals and the popular press. Special focus is on the fructose moiety within sugars due to the former’s unique metabolic and endocrine response. Fructose-containing sugars that are ‘added’ to our diets are alleged to be an important risk factor for the development of obesity [1], cardiometabolic disease [2] including metabolic syndrome [3] and diabetes [4]. Leading organizations including the World Health Organization (WHO) [5] and Canadian Diabetes Association (CDA) [6] have recommended a reduction in added sugar. The case against the harms of fructose-containing sugars appears straightforward at first, but in reality the debate is more nuanced. It was just in recent past that saturated fat was of public health concern for its adverse effects on obesity and cardiovascular disease—though the blame has now been shifted to fructose-containing sugars [7, 8]. In this paper we aim to present a review of the highest level of evidence on dietary sugars and their effect on obesity and cardiometabolic disease including important considerations that sheds light on some of the controversies around this topic.

## Historical roots

The story of dietary sugars and their potential harms on our health has oscillated several times over the past five decades as more scientific evidence on this topic came to light. Hence, it is important to give a brief historical background to the scientific controversy surrounding dietary sugars. The sugar debate first started in the 1970s when opposite positions were presented as explanations for the epidemic of cardiovascular disease. In the 1970, the American biochemist Ancel Keys, using his seven countries ecological study, argued for a role of saturated fat in heart disease [9], while in 1972 John Yudkin, a British nutritionist, warned in his book, ‘Pure, White and Deadly’ that dietary sugars were responsible for the rise in heart disease and diabetes [10]. At that time, the fat hypothesis gained general acceptance, and for the next four decades, low-fat dietary advice became part of many national nutritional guidelines with the aim of reducing the risk of chronic diseases like cardiovascular disease [11].

Dietary sugars came into forefront again in 2004 when Dr. George Bray, an eminent obesity researcher, published an ecological study that showed a parallel rise between overweight/obesity and fructose-containing sugars in the USA [12]. In an ecological study, the units of analysis are populations or groups of people rather than individuals; therefore, any conclusions derived may not apply to individuals; to do so erroneously is known as ecological fallacy [13]. It is also well recognized that an ecological study represents a very weak level of evidence [14], and Dr. Bray, rightly, presented his findings as hypothesis generating rather than causal.

Dr. Bray’s study sparked a real interest in this field and kick-started a new sugar debate that has continued fiercely to this day. Dr. Robert Lustig, a paediatrician from University of California San Francisco, in 2009 made a passionate case against fructose in the popular YouTube video, ‘Sugar: The bitter truth’ [15] that currently (November 2016) has more than six-and-a-half million views. This YouTube video was followed by a steady increase in the number of editorials, commentaries and opinion pieces in scientific literature that denoted added fructose and its related sugars [sucrose and high-fructose corn syrup (HFCS)] as health hazards while calling for measures to restrict their intake [1, 16–20]. In the past several years traditional and social media caught up with the scientific debate and have now published numerous popular books, mainstream documentaries and newspaper headlines portraying fructose-containing sugars as being toxic [21–23]. The public gets a confusing message as several nutrition researchers stand in opposite camps on the harms of fructose-containing sugars debate [24, 25]. Controversies regarding ties of researchers with the sugar industry have further fuelled the debate, e.g. it was recently reported that when writing an influential review paper that downplayed the harms of sugars several decades ago, the authors did not disclose their ties with the sugar industry [26]. It is debatable whether these ties bias the work that researchers perform; however, existence of such ties has turned a purely scientific dialogue into an intensely emotional one. It is clear that the message of the harms of sugars is much louder and dietary sugars are now squarely blamed for the rising epidemic of obesity and chronic disease in the Western nations, and with some even drawing strong parallels with tobacco [27]. The question that remains is whether, based on the best available scientific evidence, this clarion call against dietary sugars is justified?

At first glance, the case presented against sugars is quite persuasive, which hinges on implicating the fructose moiety for its unique metabolic and endocrine responses. Fructose is present in all main dietary sugars except for milk. Proponents of this fructose-centric view present ecological data, supporting it with animal studies and human mechanistic studies that show adverse effects of fructose in very high doses [28]. This evidence is backed by sound biochemical plausibility which indicates that fructose, unlike glucose, acts as an unregulated metabolic de novo substrate for fatty acid synthesis in the liver as it bypasses the main rate-limiting steps of glycolysis [29]. A new hypothesis of metabolic syndrome that involves fructose-induced increases in uric acid via the depletion of intracellular adenosine triphosphate further supports the plausibility of fructose’ special metabolic role [30]. Other likely mechanisms of harm relate to fructose’ unique endocrine signature, whereby fructose does not stimulate insulin, or the ‘satiety hormone’ leptin, nor suppresses the ‘hunger hormone’ ghrelin, leading to an overall impaired satiety signalling [31]. Functional magnetic resonance imaging (fMRI) studies suggest that fructose differentially stimulates hypothalamic centres associated with the regulation of food intake and reward compared with glucose [32]. Other mechanisms may relate to hedonic pathways, whereby fructose-containing sugary foods are over-consumed owing to their high palatability and an inability to compensate for the energy consumed in liquid form, such as sugar-sweetened beverages (SSBs) [2, 33].

It is worth noting that the bulk of the biochemical and metabolic evidence presented against fructose is based on rodent models [4, 30, 34] and human mechanistic studies [4, 32, 35]. As reviewed recently by Van Buul et al. [36], these human mechanistic studies are insufficient to demonstrate a causal role of fructose in metabolic diseases as these often involve feeding large amounts of pure fructose without concomitant glucose intake. However, fructose is commonly ingested in an almost 1:1 ratio with glucose, e.g. as the table sugar sucrose, which is a disaccharide made of the monosaccharides fructose and glucose; as fructose and glucose in high-fructose corn syrup (HFCS) found in SSBs; as fructose, glucose and sucrose found in fruit and honey. In parallel, ecological observations that link increase in fructose availability with increase in obesity, diabetes and hypertension [3, 12, 37] are offered as compelling proof that these mechanisms are indeed in operation. Even so, animal and ecological studies should only be considered to be hypothesis generating owing to their indirectness and many potential sources of bias. Moreover, extrapolation of the above mechanisms from animal models to humans needs to be done carefully, with the understanding of limitation due to biochemical and physiological differences that exist [38]. In the case of dietary sugars, it is still unclear whether the high doses given in animal models apply to the median level of fructose consumption in humans, and whether they then lead to meaningful downstream outcomes. For example, there are differences between rodents and humans on how fructose is metabolically handled in the liver. Typically, in the human liver, 50% of a fructose load is converted into glucose, 25% into lactate and approximately 15% into glycogen [39]. Stable isotope tracer studies have found that de novo lipogenesis pathway for fructose in humans is very minor (<1%) at moderate intake and up to 5% in overfed state, but in mice livers de novo lipogenesis pathway converts typically ~30% of fructose to triglycerides and reaches beyond >50% in overfed states [36, 40].

Several international and national health authorities have recently changed their dietary guidelines recommending a restriction in dietary sugars intake. Is this recommendation based on evidence of harm in relation to obesity, diabetes and cardiovascular disease? A careful review of these guidelines reveals that most of these guidelines use evidence from dental harms and caloric harms associated with excess sugar, especially SSB intake. The WHO recommended added sugars to be <10% of total daily energy intake based on observational data from dental caries [5]. This amounts to <50 g (12 teaspoons) of added sugars/day based on a typical 2000-kilocalorie diet. The term ‘free sugars’ (we use the equivalent term ‘added sugars’ throughout this paper) is defined by WHO as ‘monosaccharides and disaccharides added to foods and beverages by the manufacturer, cook or consumer, and sugars naturally present in honey, syrups, fruit juices and fruit juice concentrates’ [5]. This definition does not include ‘intrinsic sugars’, which are those ‘incorporated within the structure of intact fruit and vegetables’ or ‘sugars from milk (lactose and galactose)’. Dietary Guidelines Advisory Committee (DGAC) that informs the US Department of Agriculture (USDA) dietary recommendations provided a similar recommendation to reduce calories from added sugars to <10% based on dietary pattern modelling [41]. Scientific Advisory Committee on Nutrition (SACN) of the UK (UK) used clinical trial data to show that high sugar intake was associated with high energy intake—so both need to be reduced—and recommended <5% intake of added sugars [42]. The Heart and Stroke Foundation of Canada followed the WHO recommendation, but in addition to citing evidence from dental caries, it also assessed evidence indicating that excess sugars (using SSB data from prospective cohort studies) consumption is associated with adverse health effects including heart disease, stroke, obesity, diabetes, high blood pressure, cancer to support a recommendation of <10% dietary intake of energy from sugars [43]. Similarly, using evidence from prospective cohort studies of SSB intake on risk of obesity and diabetes, the CDA also submitted a position statement in September 2015 recommending <10% calories to come from added sugars [6]. The Nordic Nutrition Recommendations of 2012 also called for a restriction of added sugars to be kept below 10% total energy intake [44]. The evidence used in this case was threefold: (1) restriction on added sugars will ensure adequate intake of micronutrients and dietary fibre while supporting a healthy dietary pattern, (2) SSBs are associated with an increased risk of type-2 diabetes and excess weight gain and should, therefore, be limited, and (3) avoidance of frequent consumption of sugar-containing foods can reduce risk of dental caries. It is worth noting that in all of the above guidelines, the major focus was on restricting calories from added sugars especially in the form of SSBs, which suggests that more consideration was given to calories rather than any unique biochemical signal from fructose-containing sugars. Indeed, there was no restriction on fruits and vegetables which also contribute to fructose-containing sugars in the diet.

Dietary sugars intake has become a charged and emotive issue, both academically and in the media, and it is essential that we separate statements and opinions from the clinical evidence produced by high-quality research studies. Careful consideration should be given to the totality of evidence in this debate including the highest level of evidence from human clinical studies. It seems premature to provide human mechanistic studies as proof of harm in the absence of clear evidence of adverse effects of fructose on clinically meaningful outcomes. The major question that needs to be answered is: is there something special about fructose metabolism that increases the risk of obesity and chronic disease, or is the harm is related just to the excess calories it provides. In this review, we aim to present a synthesis of the highest quality evidence involving human subjects to answer this question.

## Level of evidence

**Fig. 1:**
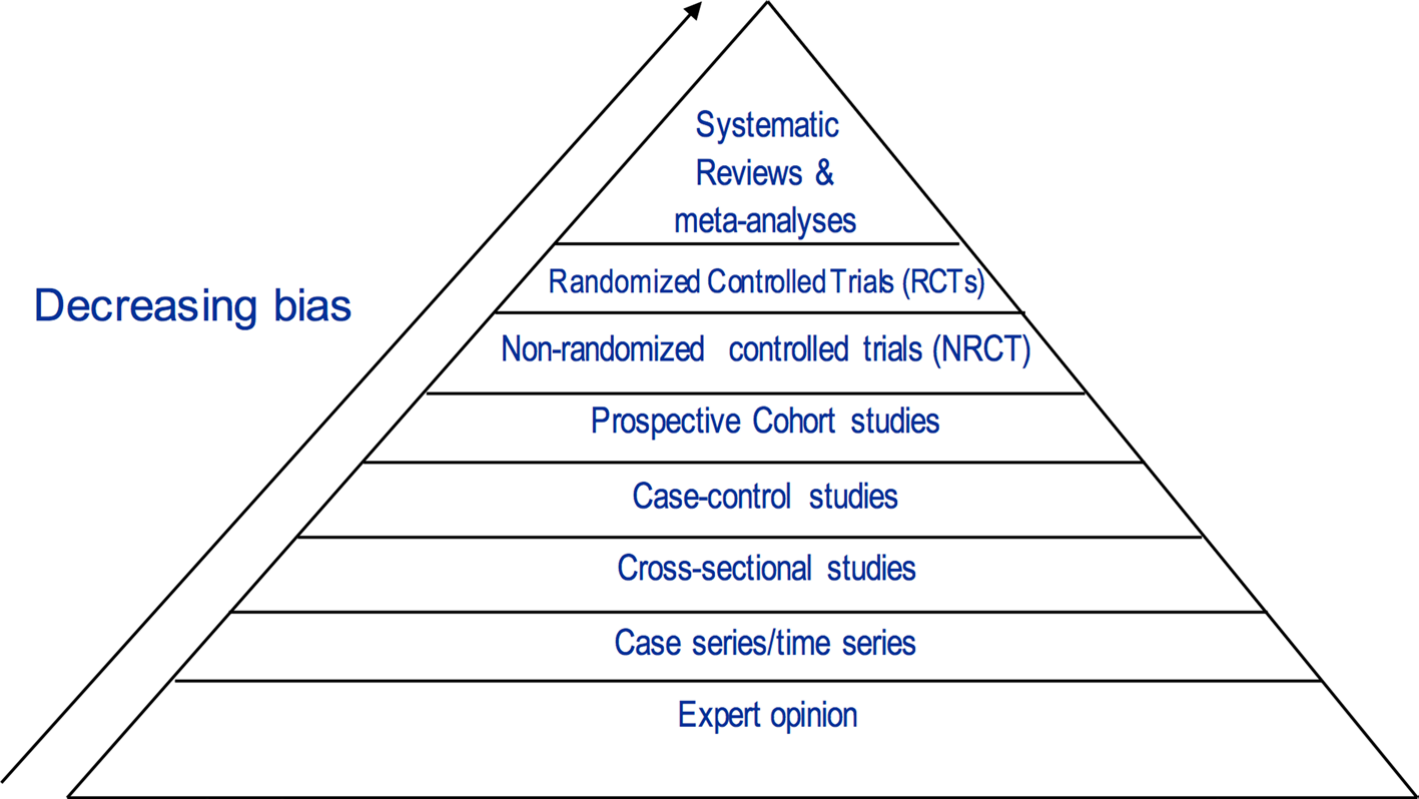
Hierarchy of evidence in evidence-based medicine

Figure 1 shows the hierarchy of evidence, initially developed by the Canadian Task Force on the Periodic Health Examination to help decide on priorities when searching for studies to answer clinical questions [45] and was subsequently adopted by the US Preventive Services Task Force [46]. This evidence-based framework represents a universally recognized accepted standard that informs public health policy and clinical practice guidelines, and it shows that the best source of evidence comes from randomized controlled trials (RCTs) because they offer the best protection from bias [47]. The only level of observational evidence that informs public health policy and clinical practice guidelines is prospective cohort studies, and we will start our presentation from this level of evidence.

## Evidence from prospective cohort studies

Prospective cohort studies are characterized by a long-term longitudinal follow-up, good measurement of dietary exposures, superior ascertainment of incidence of disease and mortality outcomes (and not just surrogate markers) and the ability to adjust for multiple confounding factors [48]. These advantages enable prospective cohort studies to present some of the strongest evidence from observational studies for assessing the relation of dietary sugars exposure to obesity, diabetes and cardiovascular disease. While this makes prospective cohort studies a good place to look at the question of sugars and incidence of disease, drawing clear inferences from prospective cohort studies is somewhat complicated by the form in which fructose-containing sugars are consumed as the majority of evidence is available only from SSBs.

A WHO-commissioned systematic review and meta-analysis of prospective cohort studies did not find any association between total fructose-containing sugars and body weight [49]. Similarly, large prospective cohort studies have not shown any association with diabetes [50], hypertension [51] and coronary heart disease (CHD) [52]. One exception is gout where it was associated with fructose consumption in a systematic review and meta-analysis of prospective cohort studies [53].

Regular consumption of SSBs indicates that it can lead to weight gain and substantially increase risk of developing cardiometabolic diseases [54]. A WHO-commissioned systematic review and meta-analysis of 38 prospective cohort studies showed a significant association between SSBs and the risk of overweight/obesity in children and weight gain in adults [49]. Another meta-analysis of 22 prospective cohort studies found similar results in both children and adults [55]. In like manner, a systematic review and meta-analysis of 17 prospective cohort studies showed a similar adverse association between SSBs and incident diabetes [56]. Significant relationship of SSBs also exists with metabolic syndrome [57], hypertension [58], CHD [59, 60], stroke [61] and gout [62].

**Fig. 2:**
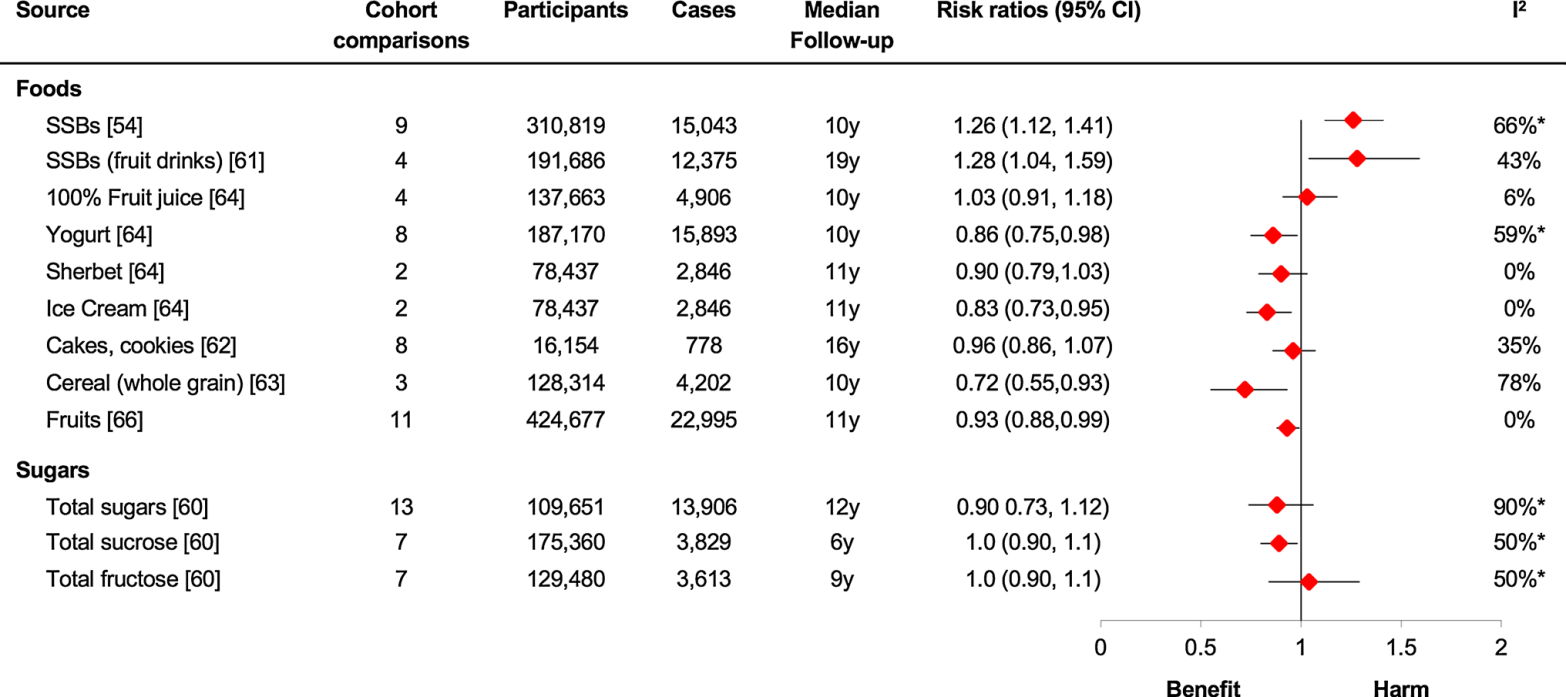
Sources of sugars and incident type 2 diabetes. Adapted from [68]. Summary estimates (*diamonds*) were derived from pooled risk ratios for comparison of extreme quantiles (the highest level of exposure compared with the lowest level of exposure). The one exception was for cakes and cookies, which compared the highest level of exposure with the middle level of exposure; the reference exposure that was associated with the lowest risk. Data are expressed as risk ratios with 95% CIs. *Asterisks* indicate significant interstudy heterogeneity as assessed by the Cochran *Q* statistic and quantified by the *I* ^2^ statistic at a significance level of *P* < .10. *SSBs* sugar-sweetened beverages

Meanwhile, pooled analyses involving many of the same prospective cohort studies that were used in the analysis of SSBs did not find harmful relationships between type 2 diabetes and total dietary sources of sugars (liquid or solid), total sucrose, total fructose [63] or other sources of added fructose such as 100% fruit juice [64] and cakes and cookies [65]. In fact, some sources of sugars including whole-grain cereals [66], yogurt [67] and ice cream [67] showed a positive benefit with type 2 diabetes (see Fig. 2). This does not necessarily mean that ice cream might be beneficial for type 2 diabetes, but highlights the complexity of the relationship between the dietary source of sugars and disease outcomes [68]. To make matters even more complex, another important source of fructose-containing sugars, fruits and vegetables, has consistently shown to reduce the risk of type 2 diabetes [69], CHD and total mortality [70] in pooled analyses of large prospective cohort studies.

## Are sugar-sweetened beverages a special case?

The contrast of results between sugars provided by SSBs and sugars from other food sources is striking; the former shows a consistent signal of harm, while the latter shows either no harm or a benefit. These results suggest that sugars from SSBs might be a special case and this assertion is supported by several plausible explanations. One, the association might be related to energy as the observed associations between SSBs and cardiometabolic diseases only remain significant at the highest quantiles of exposures and do not remain significant at mean levels of exposures for USA, the exception being gout [53]. The disappearance or marked attenuation of the effect of SSBs on weight gain after adjustment for total energy in one meta-analysis [55] and on diabetes risk after adjustment of adiposity [56], a proxy of high caloric intake, in another meta-analysis suggests that the effect of SSBs on cardiometabolic diseases appears to be highly mediated by energy. Two, it is possible that liquid calories from SSBs beverages are poorly compensated for by a decrease in total energy intake compared to solid calories, leading to weight gain and downstream cardiometabolic diseases. This hypothesis, however, remains unproven compared to acute preload trials which show that liquid calories were less compensated than solid calories [33]. Long-term trials on this subject designed to assess whether this lack of compensation results in weight gain have been inconclusive [71, 72]. In contrast to SSBs, liquid calories from 100% pure fruit juice have also not shown reliable associations with diabetes or cardiometabolic diseases [56, 64]. Three, SSBs are easier to measure in research studies, e.g. number of cans of sweetened beverages like Coca-Cola can be recalled with precise fidelity, which is not the case for other sources of fructose-containing sugars. The latter are usually estimated from individual foods leading to increased measurement error due to the imprecise nature of portions and the actual content of sugars. Four, compared to SSBs, others sugar sources include nutrient-dense fruits and vegetables as well as whole-grain products that contain potential health-enhancing nutrients, fibre and phytochemicals. In fact, these sources of sugars have been associated with weight loss and improved metabolic outcomes in large prospective cohort studies [69, 70, 73] and randomized dietary trials [74, 75] and, as such, may balance any harms associated with sugars. Five, SSBs might be a marker of an unhealthy lifestyle. Individual foods and lifestyle choices do not exist in isolation, and an approach that only looks at them individually may be inadequate for disentangling complicated interactions between different foods and dietary and lifestyle patterns in real-world situations. In this regard, it has been shown that sugar-sweetened beverage consumers are different from those who do not consume sugary drinks; the former snack more, consume more calories, smoke more, exercise less and have a worse dietary pattern [59, 76, 77]. Such lifestyle and dietary factors are generally adjusted for in the analyses of prospective cohort studies as they may confound the association between sugar-sweetened beverages and disease outcomes. However, it is well recognized that complete correction of such confounding variables is not possible due to residual confounding. Such variables might not be measured, measured imprecisely or not adjusted at all as the total number of confounders is unknown [78–80]. While these important collinear effects between such lifestyle factors and SSBs might bias the results in prospective cohort studies, such collinear effects can be exploited to an advantage by taking a larger snapshot using dietary patterns.

Analysis of food consumption as dietary patterns offer a more comprehensive approach to the relationship between diet and disease and as such might shed a light on what foods, which might in themselves be associated with disease, are taken along with SSBs. The Harvard cohorts (Nurses’ Health Study, the Nurses’ Health Study II and the Health Professionals Follow-up Study) have revealed two distinct dietary patterns. One is the Western dietary pattern, which is characterized by high intakes of SSBs, processed meats, red meats, refined grains, French fries, sweets and desserts, and the second is the prudent dietary pattern characterized by high intakes of vegetables, fruit, legumes, fish, poultry and whole grains. The Western dietary pattern is associated with increased cardiometabolic disease outcomes that includes weight gain, increased risk of diabetes, CHD and mortality resulting from CHD in energy-adjusted models [81–83]. In fact, the weight gain associated with a Western dietary pattern [83] appears to be greater compared to the weight gain associated with SSBs alone [84, 85]. Each individual food component of the Western dietary pattern also has a greater relative risk of diabetes (RR = 2.56–2.93) [86–89] and CHD (RR = 1.46) [90] than those reported for SSBs alone (RR = 1.01–1.18, per serving) [49, 55–60]. Moreover, these effects on bodyweight of the Western dietary pattern persist even after adjustment for sugar-sweetened beverage intake in studies in which this adjustment was made [83, 86] suggesting that the Western dietary pattern as a whole is a more important factor contributing to increased energy intake and weight gain, compared to SSBs alone.

There are some important caveats to the clear and consistent relationship between SSBs and cardiometabolic disease. The associations for SSBs are only significant when comparing extreme comparisons, i.e. typically ≥1–2 servings/day versus none or <1 serving/month, or an increase in energy consumption from each additional serving/day. No significant associations have been seen at moderate levels of intake [49, 59, 61, 62, 91], which are around the mean estimated global intake of SSBs 0.58 servings/day [92] or approximately 15 g of added sugars/day.

How does a moderate consumption of SSBs contribute to the burden of disease at a global level? The recent Global Burden of Disease Study used national level data on 67 measured risk factors for disease including SSB consumption worldwide [93]. The authors estimated global, regional and national disease burdens using best available estimates of the association of each risk factor with obesity, diabetes mellitus, cardiovascular disease and cancers, and combined it with age-, sex- and cause-specific mortality using a comparative risk assessment analytic framework [94]. For SSBs, these estimates included both direct effects on disease burden but also indirect effects via increased obesity. When looking at the ranking of the top 15 dietary and physical activity factors, high intake of SSBs was ranked only 12th for its burden on total global mortality and was the only risk factor with added sugars [93]. High blood pressure, tobacco smoking, household air pollution, diet low in fruits and alcohol use were ranked 1–5, respectively, as the leading global risk factors for attributable burden of disease. In comparison, high intake of SSBs was ranked all the way down at number 32nd globally for attributable burden of disease. When counting total estimated deaths, the authors found that in 2010, dietary risk accounted for an estimated 11.3 million deaths worldwide [93], but SSBs only accounted for an estimated 184k deaths (1.6% of total deaths attributed to dietary risk) [94]. In only a few countries SSBs accounted for a much larger share of disease burden due to higher average consumption, e.g. Mexico had the largest absolute (405 deaths/million) and proportional mortality attributable to SSBs (12.1%), with one of the highest mean intakes (2.6 servings/day), but such a high intake is an exception and not the norm across the globe as 90 per cent of countries had an average SSB intake of <1 serving/day [92]. USA also had a relatively high average consumption of SSBs (1.3 servings/day; 42.9 g/day added sugars) but that contributed only 2.3% of proportional mortality [94]. This study underscored that compared to other risk factors, SSBs play a relatively minor role in global disease burden.

If we take a look at the potential risk of individual diseases, the effect sizes associated with SSBs tend to be relatively modest. For example, pooled relative risk (RR) for SSBs with cardiometabolic diseases including metabolic syndrome (RR = 1.20, highest vs. lowest quantile) [57], diabetes (RR = 1.18, per serving increase) [56], hypertension (RR = 1.12, highest vs. lowest quantile) [58], CHD (RR = 1.17, highest vs. lowest quantile) [60], stroke (RR = 1.06, per serving increase) [95] is relatively modest and did not exceed 1.20. Focusing on type 2 diabetes, the risk estimate of SSBs intake is similar to or even lower than those of other established dietary risk factors, processed meats (RR = 1.51, 100 g/day) [96], red meat (RR = 1.19, 50 g/day) [96, 97], French fries (RR = 1.16, two servings/week) [98]; high glycaemic index (RR = 1.33, highest vs. lowest quantile) [99], fried food (RR = 1.55, <1 vs. >7 time a week) [100] and potatoes (RR = 1.18, one serving/day) [98]. When we look beyond dietary factors, the association of SSBs with type 2 diabetes pales in comparison. The association of body mass index (lowest versus highest levels) with type 2 diabetes ranges from 10 to 30 times [101], which is similar to risk levels associated with long-term smoking and lung cancer [102]. As these risk ratios are several fold higher than the risk estimates seen with SSB intake, the analogy that SSBs are to obesity and diabetes as cigarette smoking to lung cancer [27, 103, 104] does not stand. Thus, the evidence suggests that similar to other dietary factors, the association of SSBs with diabetes is modest and it cannot be singled out and put in the bracket of other non-dietary factors.

**Fig. 3:**
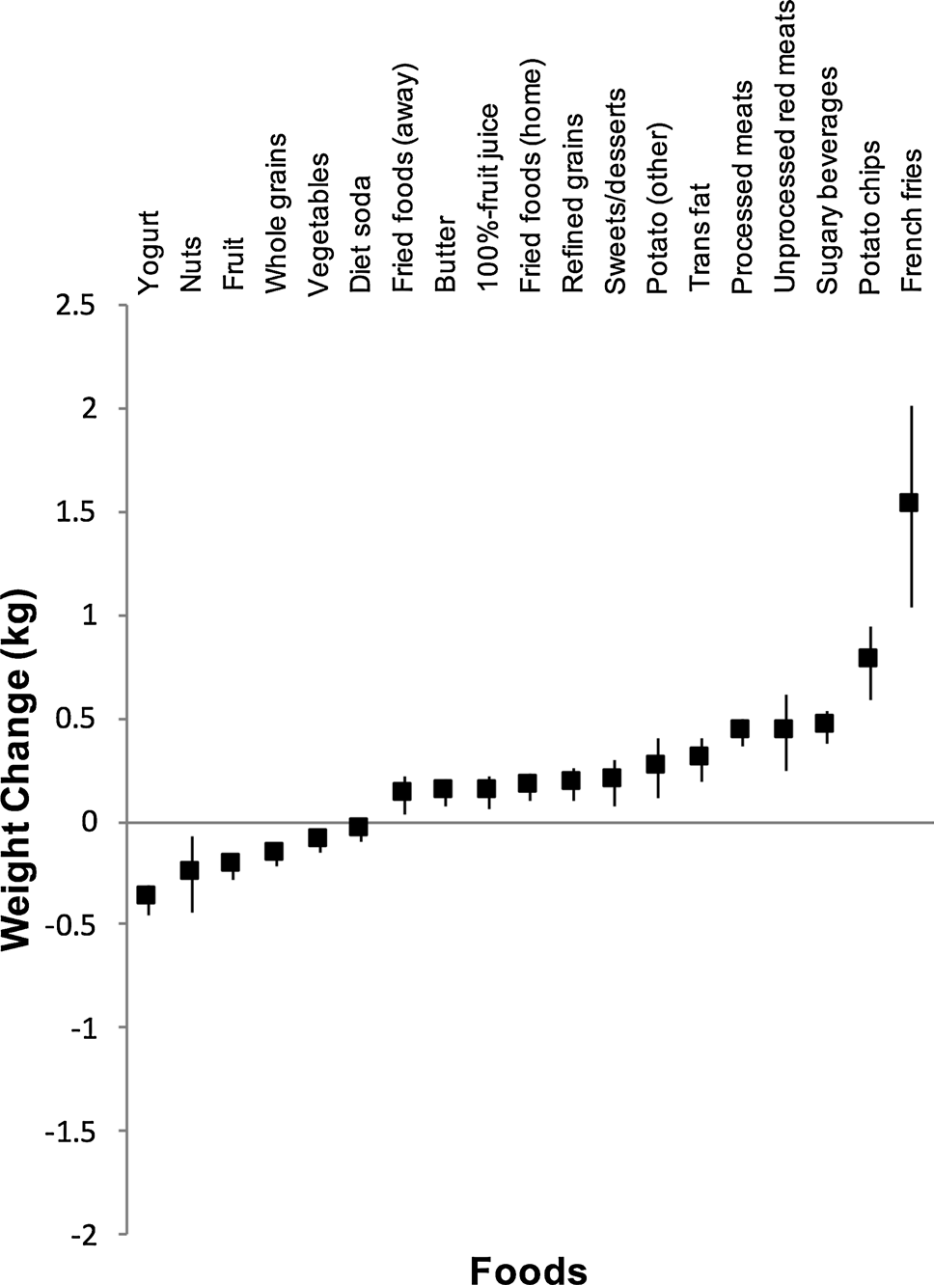
Bodyweight changes (kg) over a 4-year period associated with an increase in the consumption of different food items. Using data from the Nurses’ Health Study, the Nurses’ Health Study II and the Health Professionals Follow-up Study as reported by Mozaffarian et al. [105]. Increased consumption is based on servings/day for all items except trans fat (per cent total energy) and fried foods (servings/week). Data represent pooled mean changes with 95% confidence intervals adjusted for age, baseline body mass index at the start of each 4-year interval, sleep duration and changes in physical activity, smoking, alcohol use, television watching and each additional food item

The relatively modest risk ratios of the association of per serving increase in SSB intake with cardiometabolic diseases begs the question, if other sources of calories in the diet are more important than SSBs? A pooled analyses of the three Harvard cohorts by Mozaffarian et al. [105] found that the weight gain observed for every 4 years of follow-up for increasing one serving of sugary beverages was smaller than or in range of increasing one serving of several other foods such as French fries, potato chips, unprocessed meat, processed meat, trans fat or boiled, baked or mashed potatoes when not adjusted for energy (see Fig. 3). It is likely that extra calories are driving this association of SSBs with weight gain, which suggests that SSBs provide fewer calories than the above foods.

Overall, the evidence we presented suggests that SSBs have comparatively small effect sizes seen only at the extremes of intake, which makes them an important source of excess sugary calories but not something that unusually contributes to increased cardiometabolic risk compared to many other aspects of the diet when we consider their mean intake globally.

## Evidence from controlled trials

High-quality evidence from randomized and non-RCTs offers the best protection from bias as these control for confounding factors while allowing for the isolation of the effect of interest. The major limitation of nutrition trials of dietary sugars is the lack of clinical outcomes as these require long-term follow-up over years and most nutrition trials last only a few weeks. However, risk factors or surrogate outcomes measured at intermediate times can be explored with good precision, thereby providing insight into processes that might lead to disease.

## Fructose and cardiometabolic risk factors

We can use two types of trial designs together to disentangle the direct association of various risk factors with fructose intake from the energy it provides. One, substitution trials, in which comparisons are matched for energy, with fructose, in liquid or mixed format, is substituted for other sources of carbohydrates in the diet. The substitution trials can provide information on the usefulness of low-sugar foods on shelves, which are usually backfilled with other carbohydrates including starch and maltodextrins, so in many cases the total amount of calories do not change [106]. The second type of trials is addition trials, in which comparisons are supplemented with excess energy from fructose compared with the same diet alone without excess energy, i.e. they are hypercaloric. These trials together provide information on whether it is the excess energy from fructose that leads to adverse effects.

**Fig. 4:**
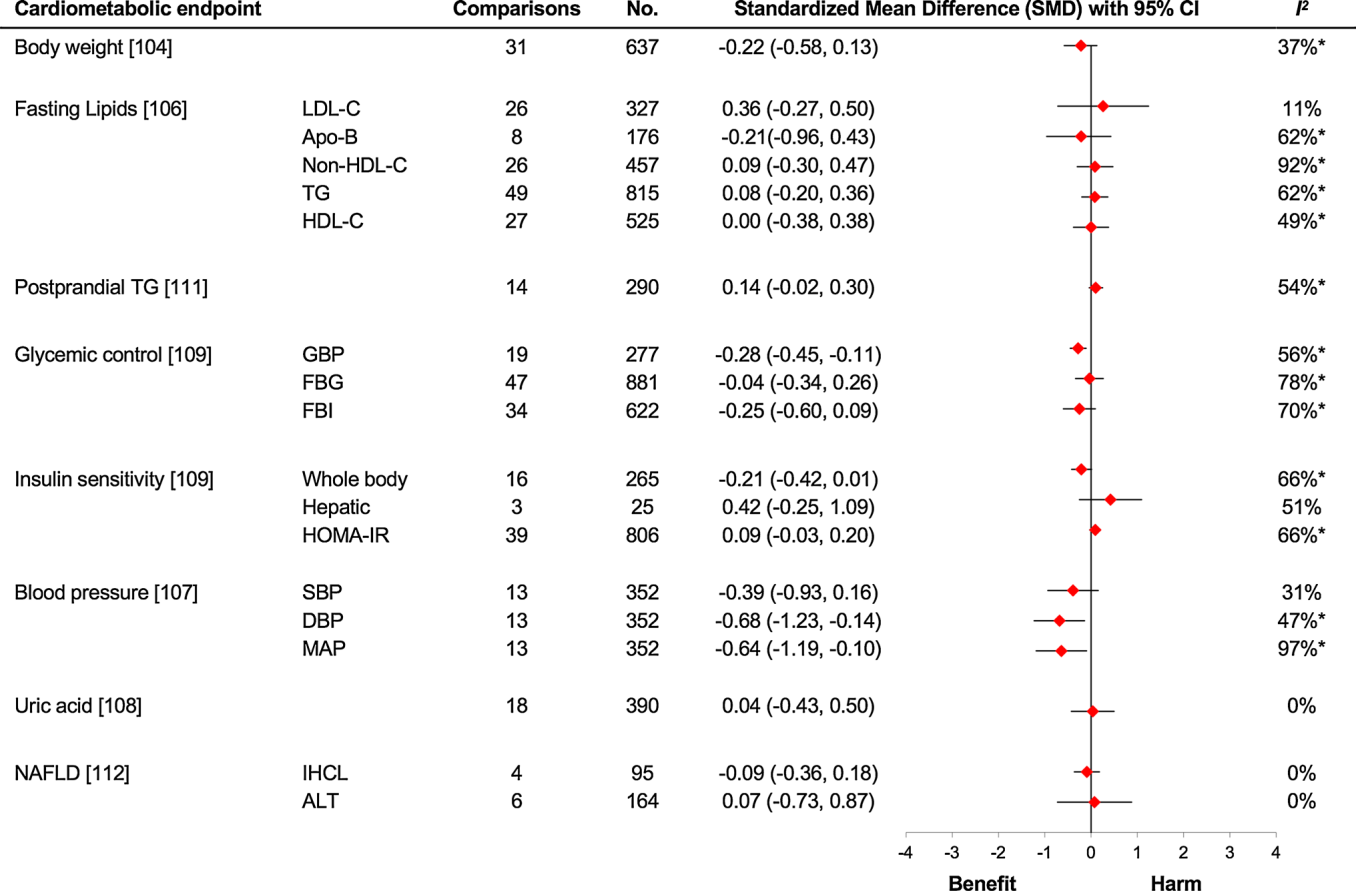
Substitution trials. The meta-analyses are of isocaloric substitution trials, in which fructose was exchanged for other carbohydrate sources under energy-matched conditions. Summary estimates (*diamonds*) were derived from pooled trial-level data. To allow the summary estimates for each endpoint to be displayed on the same axis, mean differences were transformed to standardized mean differences (SMDs). Pseudo-95% CIs for each transformed SMD were derived directly from the original mean difference and 95% CI. The scales were also flipped for high-density lipoprotein cholesterol (HDL-C), whole-body insulin sensitivity and hepatic insulin sensitivity so that the direction of the effect for benefit or harm was in the same direction as that for the other endpoints. *Asterisks* indicate significant interstudy heterogeneity as assessed by the Cochran *Q* statistic and quantified by the *I* ^2^ statistic at a significance level of *P* < .10 (the higher significance level was chosen owing to the poor sensitivity of the test). *ALT* alanine aminotransferase, *Apo*-*B* apolipoprotein B, *DBP* diastolic blood pressure, *FBG* fasting blood glucose, *FBI* fasting blood insulin, *GBP* glycated blood proteins, *HOMA*-*IR* homoeostatic model assessment-insulin resistance, *IHCL* intrahepatocellular lipid, *LDL*-*C* low-density lipoprotein cholesterol, *MAP* mean arterial pressure, *SBP* systolic blood pressure, *TG* triglycerides, *No.* total number of participants included in the meta-analysis of the controlled dietary trials

In a Canadian Institute of Health Research (CIHR) funded series of systematic reviews and meta-analyses of controlled trials consisting of more than 50 trials and an excess of 1000 subjects, we examined the effect of fructose on various cardiometabolic risk factors when it was provided in isocaloric substitution for other carbohydrates (see Fig. 4). In the pooled analysis of the substitution trials we found no adverse effect on body weight [107], fasting lipids [108, 109], blood pressure [110], uric acid concentration [111], glycaemic control and insulin sensitivity [112, 113], postprandial lipids [114] and markers of non-alcoholic fatty liver disease (NAFLD) [115] in individuals with varying metabolic phenotypes. In the above studies, the dose of fructose ranged from a moderate 22.5 to 300 g/day with the follow-up ranging from one to 52 weeks. In these studies, fructose shows a slight benefit for glycaemic control and blood pressure when isocalorically exchanged for other sources of carbohydrates [110, 112, 113]. The improvement in glycaemic control as assessed by glycated blood proteins was equivalent to 0.57% reduction in haemoglobin A1c [112, 113], which is at the lower limit of efficacy of oral antihyperglycaemic agents of 0.5% [116] and exceeds the US Food and Drug Administration’s threshold of 0.3% for the development of new oral antihyperglycaemic agents both in individuals with and without diabetes [117]. Furthermore, in a previous meta-analysis, the effect of fructose on HbA1c has been found to be dose dependent [118] which gives credence to the above results. In short, these substitution studies demonstrate that if we match calories with other carbohydrates, fructose is not associated with an increased harm for cardiometabolic parameters and may even be beneficial in low doses.

Conversely, the adverse effect of fructose in isocaloric trials is seen only under certain conditions. The dose threshold of harm for the effect of fructose on fasting triglycerides has been reported as >60 g/day in people with type 2 diabetes and >100 g/day in those with mixed phenotypes, and a threshold of >50 g/day for postprandial triglycerides in those with mixed phenotypes [107, 118]. From pooled and individual controlled trials, the only condition in which isocaloric comparisons of fructose were found to be associated with increase in both fasting and postprandial triglycerides was when very high doses (>100 g/day) of fructose were provided [119, 120]. However, these findings have not been reproduced in dose–response and subgroup analyses in updated systematic reviews and meta-analyses [108, 114].

Why would fructose show benefit on HbA1c or glycated haemoglobin in low doses? Fructose might be needed in smaller amounts as it is sweeter than glucose. The relative sweetness in a 10% solution for fructose is 117, glucose is 65, and sucrose is 100 [121]. Although, in nature, fructose is always present along with glucose, a median higher sweetness with fructose compared to glucose would probably not lead to larger intakes. Moreover, biological evidence suggests that fructose might assist in the metabolic handling of glucose. Fructose has a very low glycaemic index of 14 compared to glucose (=100) and sucrose (=65) [122] which might lower HbA1c levels. However, such an affect is not seen at very high dose of fructose, where it impairs insulin sensitivity driving up HbA1c [118]. The low glycaemic index of fructose also led to an early interest for its role in diabetes management. Emerging evidence also suggests that low-dose fructose (approx. 10 g/meal—equal to one apple) may benefit glycaemic control through its metabolite fructose-1-phosphate by inducing glucokinase activity [123]. In vitro studies in cultured hepatocytes demonstrate that fructose-1-P, a fructose metabolite, displaces fructose-6-P from the regulatory glucokinase-binding protein in the nucleus, causing the translocation of glucokinase to the cytosol. Glucosidase in turn increases the phosphorylation of incoming glucose and suppression of endogenous glucose production [124–127]. This catalytic effect of fructose has been reported in vivo also, where a reduction in hepatic glucose production under hyperglycaemic clamp conditions was seen in patients with type 2 diabetes and an increase in glycogen synthesis by carbon-13 nuclear magnetic resonance spectroscopy under euglycaemic clamp conditions was found in participants without diabetes [123, 128]. Similarly, these catalytic effects are also seen acute clinical studies. Catalytic doses of fructose at 7.5–10 g have shown to reduce postprandial glycaemic response to high glycaemic index meals like oral glucose or mashed potatoes in healthy participants [129, 130] and those with type 2 diabetes [131]. These mechanisms also appear to be sustainable over long-term intake of fructose. Our systematic review and meta-analysis of controlled trials on the effect of small catalytic fructose doses (≤36 g/day) in exchange for starch showed similar glycaemic benefits as those in higher doses without any adverse effects on metabolic control over 1–52 weeks of follow-up [113]. Furthermore, in our re-analysis of glycaemic control trials, fructose-containing sugars showed harm in hypercaloric trials but demonstrated a strong protective effect in isocaloric trials [132]. These findings suggest that the metabolic effect of sugars should be considered separately from their caloric effects.

**Fig. 5:**
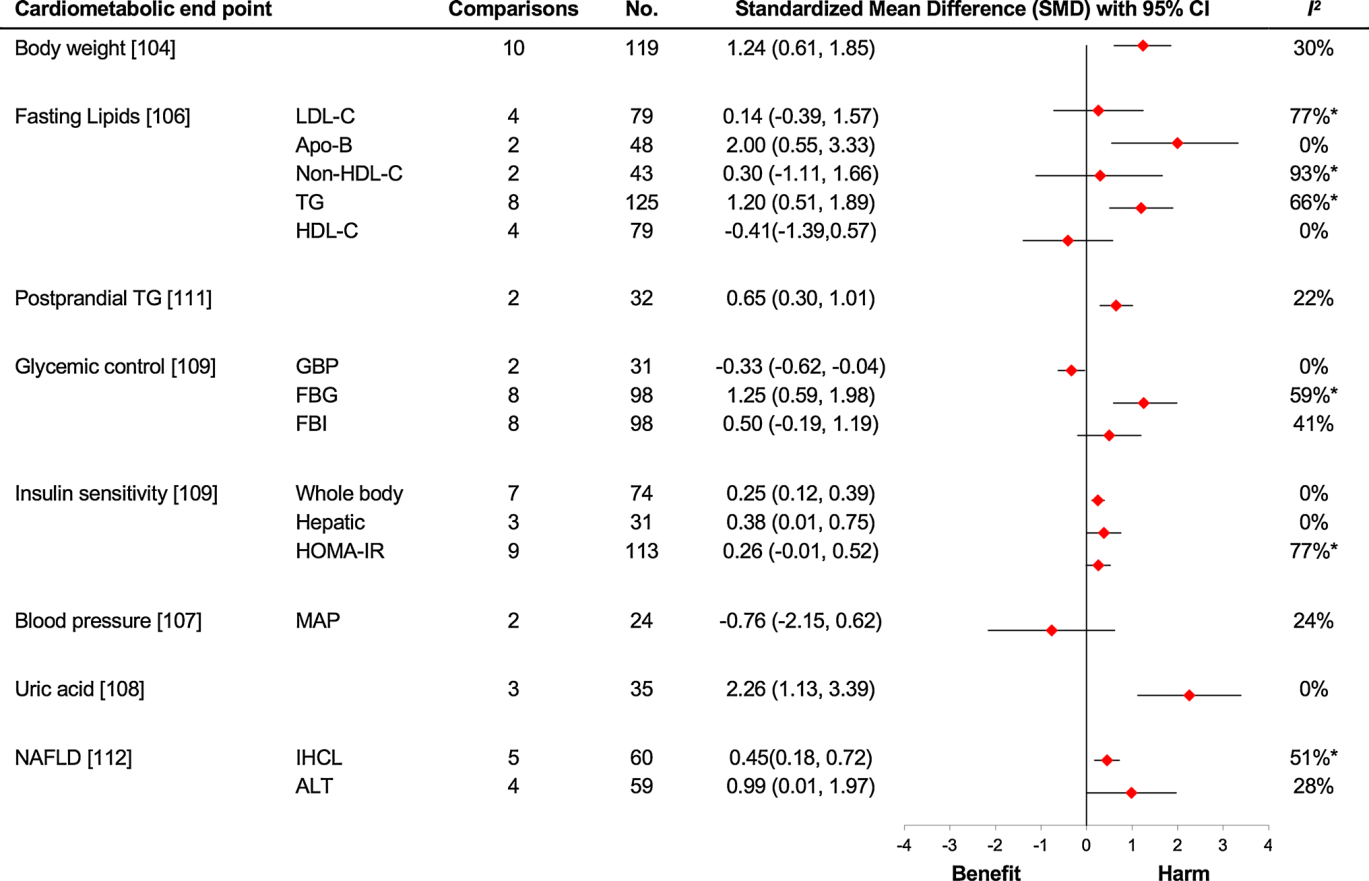
Addition trials. The meta-analyses are of hypercaloric addition trials, in which excess calories from fructose were added to a diet compared with the same diet without the excess calories. Summary estimates (*diamonds*) were derived from pooled trial-level data. To allow the summary estimates for each endpoint to be displayed on the same axis, mean differences were transformed to standardized mean differences (SMDs). Pseudo-95% CIs for each transformed SMD were derived directly from the original mean difference and 95% CI. The scales were also flipped for high-density lipoprotein cholesterol (HDL-C), whole-body insulin sensitivity and hepatic insulin sensitivity so that the direction of the effect for benefit or harm was in the same direction as that for the other endpoints. *Asterisks* indicate significant interstudy heterogeneity as assessed by the Cochran *Q* statistic and quantified by the *I* ^2^ statistic at a significance level of *P* < .10 (the higher significance level was chosen owing to the poor sensitivity of the test). *ALT* alanine aminotransferase, *Apo*-*B* apolipoprotein B, *DBP* diastolic blood pressure, *FBG* fasting blood glucose, *FBI* fasting blood insulin, *GBP* glycated blood proteins, *HOMA*-*IR* homoeostatic model assessment-insulin resistance, *IHCL* intrahepatocellular lipid, *LDL*-*C* low-density lipoprotein cholesterol, *MAP* mean arterial pressure, *SBP* systolic blood pressure, *TG* triglycerides, *No.* total number of participants included in the meta-analysis of the controlled dietary trials

Similar to rodent studies of overfeeding where we see a consistent signal for harm [34], in humans a signal for harm is found in hypercaloric addition trials in which fructose is added to provide excess calories in the diet and compared to the same diet without the excess calories. Systematic reviews and meta-analysis of such trials have shown that fructose intake providing excess calories is associated with weight gain [107], increase in fasting [108, 109] and postprandial triglyceride levels [114], fasting glucose levels and insulin resistance [112, 113], uric acid concentrations [111] and markers of NAFLD [115] (see Fig. 5). The inability of fructose to demonstrate the same adverse associations in isocaloric substitution for other carbohydrates suggests that the primary determinant of these observed harms is excess calories rather than any unusual metabolic or endocrine response to fructose. Thus, the adverse effects observed under conditions of overfeeding appear to be no more mediated by fructose than by other sources of carbohydrate used to replace it. Also, that these adverse effects appear to be reversible by exercise suggests that such adverse effects of fructose overfeeding may not be applicable to those who engage in regular physical activity [120, 133].

## Fructose-containing sugars and weight

A criticism that can be levelled on the systematic reviews and meta-analyses of controlled trials of fructose described above is that in diet pure fructose is not consumed in isolation but is commonly consumed together with glucose, either in form of HFCS or honey, or as part of the sucrose molecule. In other words, it can be argued that the above studies do not represent real-world situations for the vast majority of people. For this reason, we investigated the trials of fructose-containing sugars (HFCS, sucrose, honey, etc.) found normally in the diet and examined their effects on cardiometabolic outcomes. To allow the direct effects of fructose-containing sugars to be disentangled from calories, four types of study designs can be described: (1) substitution trials, in which fructose-containing sugars added to foods and beverages are compared with other macronutrient sources under energy-matched conditions; (2) addition trials, in which fructose-containing sugars supplemented a diet with excess energy compared to the same diet supplemented with the equivalent amounts of non-caloric food and beverages or the same diet alone without the excess energy from fructose-containing sugars; (3) subtraction trials, in which energy from fructose-containing sugars was reduced through displacement with water and/or no-calorie or low-calorie sweeteners or by eliminating it altogether from the background diet; and (4) ad libitum trials, in which energy from fructose-containing sugars was freely replaced with other foods like complex carbohydrates and fat without any strict control of either the study foods or the background diet.

**Fig. 6:**
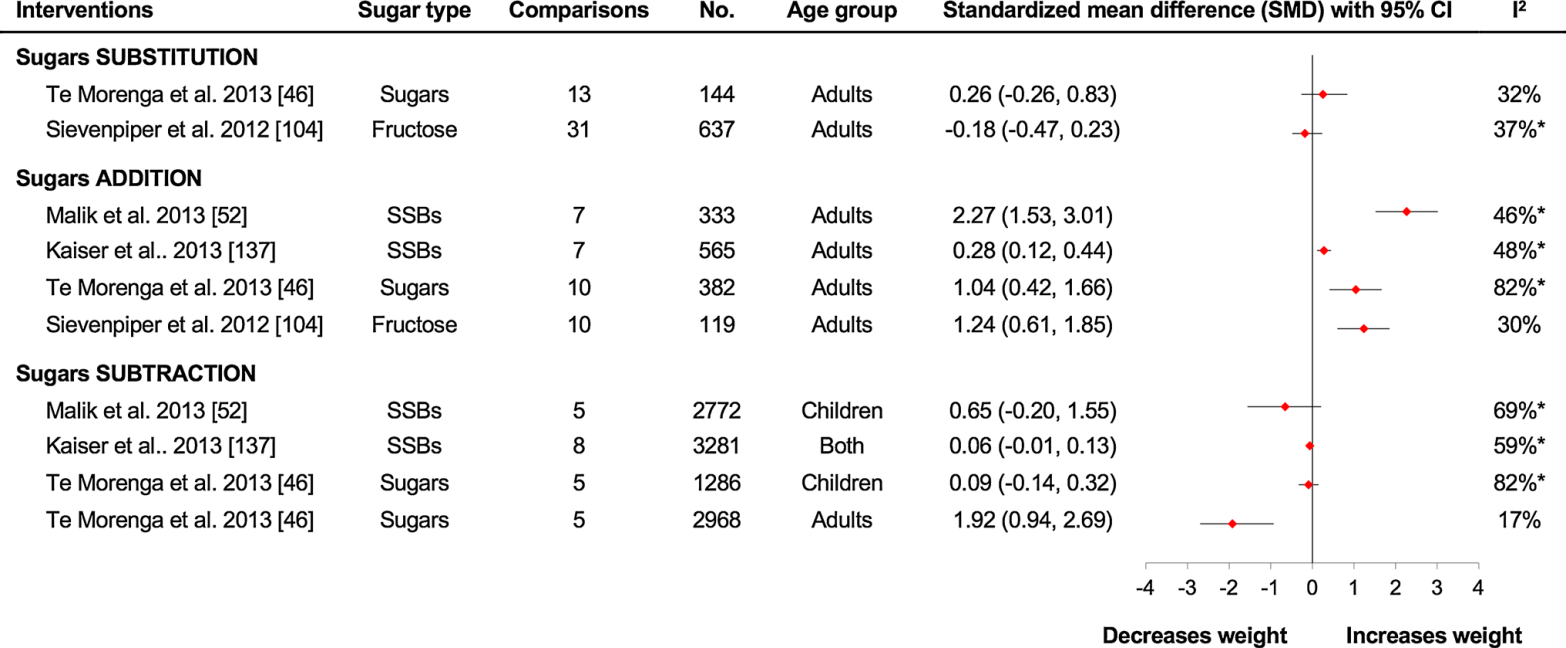
Fructose-containing sugars and weight change in controlled dietary trials. Forest plots of summary estimates from recent meta-analyses of the effect of different fructose-containing sugars interventions on indices of body weight in controlled dietary trials involving children and adults. The meta-analyses were grouped broadly based on the interventions in question: isocaloric sugar substitution interventions, in which sugars were exchanged for other carbohydrate sources under energy-matched conditions; sugar supplementation interventions, in which sugars supplement background diets providing excess energy compared with the background diets alone without the excess energy; and sugar reduction interventions, in which excess from sugars is reduced in background diets compared with the background diets still containing the sugars. Indices of body weight included body weight in Sievenpiper et al. [107], Kaiser et al. [140] and Te Morenga et al. [49]. For isocaloric sugar substitution only; body fatness in Te Morenga et al. [49] for all other comparisons; and BMI *z* scores in Malik et al. [55]. Summary estimates (*diamonds*) were derived from pooled trial-level data. To allow the summary estimates for each endpoint to be displayed on the same axis, mean differences (MDs) were transformed to standardized means differences (SMDs). Pseudo-95% confidence intervals (CI) for each transformed SMD were derived directly from the original MD and 95% CI. *Asterisks* indicate significant interstudy heterogeneity as assessed by the Cochran *Q* statistic and quantified by the *I* ^2^-statistic at a significance level of *P* < .10. *SSBs* sugar-sweetened beverages, *No.* total number of participants included in the meta-analysis of the controlled dietary trials

Two separate systematic reviews and meta-analyses of substitution trials showed no difference for fructose-containing sugars on bodyweight when they were replaced isocalorically with other macronutrient sources (usually starch or other sugars) (see Fig. 6). The first one was a WHO-commissioned systematic review and meta-analysis of 13 RCTs involving 144 adults [49]. It found that total fructose-containing added sugars (predominantly sucrose) did not affect bodyweight when substituted for other sources of carbohydrates (predominantly starch). Our own pooled analysis of 31 randomized and non-randomized controlled dietary trials assessing the effect of consumption of chronic fructose-containing sugars in substitution for other sources of carbohydrates (mainly starch) under energy-matched conditions did not support an adverse effect of fructose on body weight in 637 adults [107]. Similar effects persisted even when fructose was provided in liquid form and also under condition of positive energy balance. When we restricted the analysis to five ‘substitution’ trials with a positive energy balance but energy matched between the arms, there was still no evidence that fructose affects bodyweight differently compared to other carbohydrates.

A follow-up systematic review and meta-analysis by the same WHO group showed mixed effects of fructose-containing added sugars on other cardiometabolic risk factors [134]. In this paper the authors showed that in isocaloric comparisons with other sources of macronutrients, mainly starch, the fructose-containing sugars had beneficial effects on high-density lipoprotein cholesterol (HDL-C) but adverse effects on triglyceride levels, low-density lipoprotein cholesterol (LDL-C) and total cholesterol levels. Fructose-containing sugars had no effect on systolic or diastolic blood pressure. While the paper findings present a good case against fructose-containing sugars, there are issues which limit their generalization. One, the author’s definition of sugars included non-fructose-containing sugars including high glycaemic index sugars like glucose and maltose. Two, it is possible that the type of analysis performed did not allow separation of the effect of fructose from calories, as almost 40 per cent of the studies in people who consumed ad libitum diets had no strict control of energy intake. Three, the isocaloric trials were not strictly isocaloric as the trials did not include adequate measures of compliance. Four, there was significant unexplained heterogeneity in the trials and imprecision of the summary pooled estimates of most endpoints. Conversely, our most recently concluded systematic review and meta-analysis of controlled trials found that fructose-containing sugars in isocaloric comparisons with other sources of macronutrients (mainly starch) had a beneficial effect on HbA1c levels and no effect on fasting glucose or insulin levels [132]. Therefore, with the above mixed signals, it is difficult to draw strong inferences about harm or benefit for fructose-containing sugars when isocalorically exchanged with other carbohydrates.

How does fructose-containing sugars fare against macronutrients other than carbohydrates, e.g. fat and protein? Most studies comparing fructose-containing sugars with fat and protein used SSBs as the form of dietary sugars. Looking at weight gain, no difference in body weight or total body fat was seen when SSBs were substituted isocalorically for milk in a trial in children for 16 months [135] and in adults for 6 months [136]. The adult trial showed that high SSB consumption (1 l/day providing 106 g/day in added sugars) increased liver and visceral fat composition, total cholesterol and triglycerides which was not seen in calorically equivalent intake of milk [136]; in fact, milk was beneficial in reducing blood pressure. It should be noted that the dose of added sugars consumed from SSBs in this trial was at more than twice the average US population intake (which is 1.3 servings or 429 ml/day) [94]. Another 3-week trial in pre-diabetic adults of the substitution of SSBs with dairy milk also did not show any difference in weight [137]. Two studies under a negative energy balance comparing isocaloric substitutions found similar reductions in bodyweight for comparisons of sucrose versus protein or fat [138, 139]. Thus, the overall picture from substitution trials indicates that fructose-containing sugars do not appear to behave differently from other sources of macronutrients in contributing to weight gain and that there is no clear evidence for cardiometabolic harms as results are not consistent across studies.

Compared to substitution trials, consistent signal of harm was seen in the addition trials. Four systematic reviews and meta-analyses found that supplementing the diet with excess energy from fructose-containing sugars resulted in significant weight gain when compared with the same diets alone without the excess energy (see Fig. 6). The first one was the WHO-commissioned systematic review and meta-analysis of ten RCTs involving 382 adults [49]. The next two systematic reviews and meta-analyses were based upon SSB beverages intake. The study by Kaiser et al. [140] included seven RCTs with 565 adults, and the study by Malik et al. [55] included seven RCTs in 292 adults. Both studies found that supplementing diet with fructose-containing sugars in form of SSBs, providing from 150 to 530 kcal in excess calories over a period of 3 weeks–24 months, resulted in significant weight gain. Unsurprisingly, the weight gain achieved was proportional to the degree of excess calories added to the diet with some compensation [140]. In our own systematic review and meta-analysis of controlled trials, we found that fructose-containing sugars supplementing diets with excess calories, when compared with same diets without the excess calories, showed an adverse effect on fasting insulin but not on HbA1c or fasting glucose levels [132].

In the pooled analyses of subtraction trials in which calories in the diet provided by fructose-containing sugars (as added sugars or SSBs) are removed and replaced with water or non-caloric beverages, results have not shown a consistent associated with weight loss [49, 55, 140]. An observed benefit on weight was seen only in adults in a pooled analysis of five trials comprising of 2968 participants [49], in a meta-analysis of seven trials involving 2637 participants when restricted to overweight/obese individuals [140] and in a meta-analysis of 15 trials with 1591 participants using low-calorie sweeteners [141]. However, such benefit was not seen in two systematic review and meta-analyses restricted to children [49, 55], though the study by Malik et al. [55] involving five trials with 1298 participants did show a potential benefit of subtraction trials when limited to overweight and obese children only. Interestingly, the interventions in the subtraction trials were more modest than those in the addition trials, meaning the caloric difference between intervention and control arm was less and more representative of real-world intakes. Such modest effects and also the inconsistency in the results might be explained by a compensatory effect, in which people will compensate for a decrease of energy from one source by increasing intake of other foods or exert less energy to maintain a neutral energy balance [142]. Therefore, substantial weight loss by reducing SSB intake and thus sugars might be difficult in free-living conditions [140]. This was demonstrated by the Choose Healthy Options Consciously Everyday (CHOICE) trial, where the weight loss from strategy of reducing calories from SSBs did not differ from general weight loss advice at 6 months [143].

Few randomized trials have assessed the effect of displacing calories from sugars using no- or low-calorie sweeteners or water on cardiometabolic risk factors. The systematic review and meta-analysis by WHO group [134] of 39 trials only identified one subtraction trial, which used ad libitum sugar-free diet as the control, and it did not show an effect on total cholesterol, HDL-C or triglyceride levels in 32 hypertriglyceridemic men [144]. Our own systematic review and meta-analysis of five studies in 591 participants did not show an effect of reducing calories from fructose-containing sugars on HbA1c, fasting glucose or fasting insulin levels [132].

Ad libitum trials reflect real-world patterns to assess the effect of replacing fructose-containing sugars with other nutrients on weight gain and downstream cardiometabolic risk beyond the calories they provide. In these studies, fructose-containing sugars are freely replaced with other sources of energy in the diet without any requirement of a predetermined load nor a strict control of replacement or the background diet. We found very few studies with such design. The largest and the longest duration ad libitum trial was the CArbohydrate Ratio Management in European National Diets (CARMEN) study [145]. It compared ad libitum high-sugar diet (~55% energy carbohydrate, 29% energy sugars), an ad libitum high complex-carbohydrate diet (~51% energy carbohydrate, 19% energy sugars) and an ad libitum higher fat control diet (~46% energy carbohydrate, 21% energy sugars) in 398 moderately obese adults over 6 months. The weight loss and reduction in body fat were no different between ad libitum high-fructose-containing sugars diet and ad libitum high complex-carbohydrate diet, although there was a tendency for greater weight loss on the ad libitum complex-carbohydrate diet (−0.9 vs. −1.8 kg), possibly due to higher fibre and protein content. Another randomized study of 46 participants with metabolic syndrome following the same protocol over 6 months showed similar results [146]. In this study, those on the ad libitum high-fructose-containing sugars diet lost considerably more weight than those on the ad libitum higher fat control diet (−0.28 vs. +1.03 kg), although those on the high complex-carbohydrate diet lost the most weight (−4.25 kg). A third randomized trial in 20 normal-weight women followed over 2 weeks had a greater weight loss and lower plasma lipids in starch trial versus fat or sucrose arm [147]. Our own systematic review and meta-analysis of controlled trials showed no effect on HbA1c, fasting glucose or fasting insulin levels irrespective of comparator [132]. In short, evidence suggests that, under free-living conditions, it is possible to lose weight following an ad libitum diet where fructose-containing sugars are replaced with complex-carbohydrate diets that are higher in protein and fibre, but there might not be any clear advantages if replaced with other sources of energy especially from fat. However, the effect on other cardiometabolic markers is not clear and more evidence is needed.

## Should we target sugars

A lesson we can learn from the fat paradigm is that there can be unintended consequences of focusing singly on one nutrient. When saturated fat was deemed harmful, the industry responded by producing low-fat products, with no resultant appreciable calorie change, as in these products calories from fat were replaced with calories from other sources, e.g. starches and other sugars like maltodextrins [7]. The public perception changed as ‘low-fat’ products were deemed ‘healthy’, and a concomitant increase in availability on the supermarket shelf likely led to the overconsumption of such ‘low-fat’ products [148]. Not surprisingly, the expected reduction in cardiometabolic disease with the ‘low-fat’ food was not seen, and instead, we saw an unprecedented increase in incidence of overweight/obesity [149] and diabetes [150].

If a similar approach is taken by the industry who producing ‘low-sugar’ food products, a replay of the above scenario looks likely. Furthermore, the unique functional properties of fructose-containing sugars mean that their replacement is not as easy as it sounds. The functions provided in food products by sugars are related to their sensory (sweetness, taste and aroma, texture and appearance), physical (crystallization, viscosity, osmotic pressure, hygroscopicity, consistency/bulk, grain size and distribution), microbial (preservation and fermentation) and chemical (inversion and caramelization) properties [151]. Therefore, reducing or replacing sugar means one has to replace sugars with several ingredients in order to fulfil the above properties which, in many cases, may not result in calorie reduction [106]. Most commonly sugars are replaced with bulking agents, and most of these bulking agents also provide energy as most are carbohydrate-based, e.g. isomaltulose, sugar alcohols, maltodextrins and starch hydrolysates, and some are fat based. Thus, the calorie reduction in ‘low-sugar’ products might be negligible or in some cases might even increase [106]. Another side effect of a drastic reduction in sugars is that even good sources of sugars might be targeted like whole grains. For example, the calories per serving are the same (110 kcal/30 g) in Frosted Flakes and Reduced Sugar Frosted Flakes despite the total sugar content being 11 and versus 8 g, respectively. Despite appearing paradoxical, the replacement of sugars with refined corn starch means that glycaemic index of the flake product increases from 55 to 75 [152], and such an increase of GI at a whole diet level can potentially be associated with higher rates of diabetes [153]. Evidence from National Health and Nutrition Examination Survey (NHANES) data suggests that while at the population level in USA, consumption of added sugars has decreased in past two decades, there has been an increase in calories from other sources including other carbohydrates, protein and fats—such that average daily calories have not reduced [154].

## Summary

Despite the continuing concern regarding fructose’s unique metabolic effects, which stems from low-quality ecological studies, animal models and select human studies, the highest level of evidence from systematic review and meta-analysis does not support a direct causal relationship with cardiometabolic disease. Using the totality of the highest quality evidence from controlled feeding trials, we demonstrate that fructose-containing sugars can lead to weight gain, increase in cardiometabolic risk factors and disease only if it provides the excess calories. When the calories are matched, fructose-containing sugars do not appear to cause weight gain compared to other forms of macronutrients including complex carbohydrates, fats and protein, and in low doses fructose might even show benefit. Prospective cohort studies, which provide the strongest observational evidence, have shown an association between fructose-containing sugars and cardiometabolic risk including weight gain, cardiovascular disease outcomes and diabetes only when restricted to SSBs and not for sugars from other sources. In fact, the harmful effect of SSBs is likely driven by a collinearity with an unhealthy lifestyle as SSB drinkers consume more calories, exercise less, smoke more and have a poor dietary pattern.

In summary, there is nothing unique about the sugar, fructose. It is harmful when in excess but potentially beneficial when taken in small amounts—providing evidence that it is the excess energy that is causing harm and not some unique metabolic effect. Still, the potential for overconsumption of sugars in form of sugary foods and drinks is substantial, and targeting added sugars as a source of excess calories appears to be a prudent strategy. However, sugar content should not be seen as the sole determinant of a healthy diet. There are many other factors in the diet—some providing excess calories while others provide beneficial nutrients. We should consider the whole diet for health benefits compared to just focusing on one nutrient. In this regard, improvements in dietary patterns appear to have the greatest influence on weight gain and cardiometabolic risk and represent the best opportunity for successful intervention.
